# Osteocytes Specific GSK3 Inhibition Affects In Vitro Osteogenic Differentiation

**DOI:** 10.3390/biomedicines6020061

**Published:** 2018-05-21

**Authors:** Jessika Bertacchini, Maria Sara Magaroò, Francesco Potiì, Carla Palumbo

**Affiliations:** 1 Department of Biomedical, Metabolic Science and Neuroscience, University of Modena and Reggio Emilia, Via Largo del Pozzo 71, 41124 Modena, Italy; mariasara.magaro@unimore.it (M.S.M.); carla.palumbo@unimore.it (C.P.); 2 Unit of Neurosciences, Department of Medicine and Surgery, University of Parma, via Volturno 39/F, 43125 Parma, Italy; francesco.poti@unipr.it

**Keywords:** Gsk3, osteocytes, CHIR99021, osteoblast, differentiation

## Abstract

Osteocytes, the most important regulators of bone processes, are producers of molecules (usually proteins) that act as signals in order to communicate with nearby cells. These factors control cell division (proliferation), differentiation, and survival. Substantial evidence showed different signaling pathways activated by osteocytes and involved in osteoblast differentiation, in particular in the last decade, when the Wingless-related integration site (WNT) pathway assumed a critical large importance. WNT activation by inhibiting glycogen synthase kinase 3 (GSK-3) causes bone anabolism, making GSK3 a potential therapeutic target for bone diseases. In our study, we hypothesized an important role of the osteocyte MLO-Y4 conditioned medium in controlling the differentiation process of osteoblast cell line 2T3. We found an effect of diminished differentiation capability of 2T3 upon conditioning with medium from murine long bone osteocyte-Y4 cells (MLO-Y4) pre-treated with GSK3 inhibitor CHIR2201. The novel observations of this study provide knowledge about the inhibition of GSK3 in MLO-Y4 cells. This strategy could be used as a plausible target in osteocytes in order to regulate bone resorption mediated by a loss of osteoblasts activity through a paracrine loop.

## 1. Introduction

Bone development and its maintenance during adult life in response to systemic and local factors require orchestrated activity among osteocytes, osteoblasts, and osteoclasts in order to obtain a robust structure, mechanical activity, and skeletal/mineral homeostasis [1,2]. One mechanism of signal transduction is via cell communication through gap junctions, small transmembrane channels that allows the passage of molecules between neighboring cells [3,4,5]. This mode of cell-to-cell communication is related to molecules produced and secreted by osteocytes considered by many authors as regulators of osteoblasts and osteoclasts activity. In fact, osteocyte is considered as an endocrine cell able to produce and secrete soluble factors such as fibroblast FGF23 [6] and Sclerostin [7]. Substantial evidence shows different signaling pathway activated by osteocytes and involved in osteoblast differentiation, in particular in the last decade the WNT pathway assumed a large importance [8,9,10]. Members of the WNT family plays a key role during mineralization process. WNT ligands bind to the receptors Frizzled and LRP5/6 that in turn leads to the activation of B-catenin that translocates into the nucleus to activate target genes [11,12]. The changes in gene expression that represent a signal for the transition from osteoblast to osteocyte phenotype include down regulation of Alkaline Phosphatase and type I collagen and the up-regulation of genes including Dentin, Dentin matrix acidic phosphoprotein 1 (DMP1), Fibroblast growth factor 23 (FGF23), and Sclerostin (SOST) [13,14]. Induction of SOST is directly linked to the existence of mature osteocyte. This protein is a negative regulator of bone formation that blocks the WNT-B catenin signaling pathway by binding to the WNT co-receptors LRP5/6. One of the most important β-catenin regulators is Glycogen Synthase Kinase 3 that was identified as β-catenin specific kinase triggering β-catenin destabilization [15,16,17]. Several approaches were considered in order to block the GSK3 activity, and one of the most recent inhibitors used in different fields of study, like cancer research, immunotherapy research, and neurological diseases, is named CHIR2201 [16]. Our study hypothesized an important role of the osteocyte MLO-Y4 conditioned medium in controlling the differentiation process of osteoblast cell line 2T3. We found an effect of diminished differentiation capability of 2T3 upon conditioning with medium from MLO-Y4 pre-treated with GSK3 inhibitor CHIR2201. These results suggested that the inhibition of GSK3 can be used as a plausible target in osteocytes in order to regulate bone resorption mediated by a loss of osteoblasts activity through a paracrine loop.

## 2. Materials and Methods

### 2.1. Cell Cultures

Murine Long bone Osteocyte Y4 (MLO-Y4) were provided by Prof. Lynda Bonewald (UMKC, Kansas, MO, USA) and were grown at 37 °C, 5% CO_2_, (95% air), using DMEM (Sigma Aldrich, St. Louis, MO, USA), supplemented with 10% FBS (Euroclone) l-glutamine, and penicillin/streptomycin at 100 U/mL. MLO-Y4 cells were treated with GSK3 inhibitor CHIR99021. At the end of treatment, cells were collected, washed in PBS and stored at −20 °C. In parallel, the conditioned medium from the same samples (described above) were collected and stored at −80 °C.

The murine 2T3 osteoblast cell line was also obtained by Prof. Lynda Bonewald (UMKC, Kansas, MO, USA). Cells were plated at 1 × 10^4^ cells/cm^2^ and grown at 37 °C, 5% CO_2_, (95% air), using αMEM (Sigma, St. Louis, MO, USA) and l-glutamine, supplemented with 10% FBS (Euroclone, Milan, Italy), and penicillin/streptomycin at 100 U/mL. 2T3 cells were cultured under proliferative conditions and when the cultures reached confluence, the medium was replaced with osteogenic differentiation medium (DMEM with 10% FBS, 100 U/mL penicillin, 50 μg/mL streptomycin, 50 μg/mL ascorbic acid and 4 mM β-glycerophosphate). Cells were cultured at 37 °C and the medium was changed every two days. Where indicated, the differentiating medium was added to MLO-Y4 conditioned medium in a ratio of 1:4 (treated (+) with CHIR99021).

Viability of MLO-Y4 cells, treated with CHIR99021, was determined by the 3-(4,5-dimethylthiazol-2-yl)-2,5-diphenyltetrazolium bromide (MTT)-based colorimetric assay. The absorbance was read at OD = 570 nm. Moreover, cells viability was measured by Trypan blue staining.

MLO-Y4 Cells were treated overnight with 30 µg/mL BrdU, then stained with Mouse Anti-Bromodeoxyuridine/BrdU GFP-conjugated Monoclonal Antibody. To facilitate intracellular staining, cells were fixed with cold, 70% ethanol for 5 min; DNA was denatured with 1.5 M HCl for 30 min, and then cells were permeabilized with Triton X-100 0.1%(Sigma Aldrich, St. Louis, MO, USA) in PBS buffer. Detection of signals was analyzed by *COULTER*^®^ EPICS^®^ XL™ *Flow Cytometer*. (Becton Dickinson, Milan, Italy).

Neutralization experiments were performed on MLO-Y4 medium (treated and not with CHIR99021). Then, 2 mL of MLO-Y4 medium was incubated with anti-Sclerostin antibody (ABCAM) at concentration of 0.5 ug/mL at 37 °C for 3 h with gentle shaking.

Student’s T test was carried out to detect the difference between CHIR99021 treated cells and untreated cells.

### 2.2. RNA Extraction and Quantitative PCR

Total RNA was extracted with RNeasy kit (Qiagen, Hilden, Germany) and quantified by spectrophotometry with NanoDrop 2000 (Thermo Scientific, Waltham, MA, USA). 100 ng of total RNA were reverse-transcribed using iScript^TM^ cDNA Synthesis Kit (Bio-Rad Laboratories, Hercules, CA, USA). mRNA levels were quantitatively determined on a CFX96^TM^ Real-Time PCR Detection System using SsoFast^TM^ EvaGreen Supermix according to the manufacturer’s instructions (Bio-Rad Laboratories, Hercules, CA, USA). PCR primer sequences were: *Sost* forward primer 5′-TGAGAACAACCAGACCAT-3′; *Sost* reverse primer 5′-ACATCTTTGGCGTCATAG-3’; *Ctnnb1* forward primer 5′-TTAAGTCTGGTGGCATCC-3′; *Ctnnb1* reverse primer 5′-GTGATGGCGTAGAACAGTA-3′; *Hprt* forward primer 5′-ACCTGCTGGATTACATTA-3′; *Hprt* reverse primer 5′-CTTCAACAATCAAGACATTC-3′; *Gapdh* forward primer 5′-GGCATTGCTCTCTCAATGACAA-3′; *Gapdh* reverse primer 5′-ATGTAGGCCATGAGGTCCAC-3′. Relative gene expression values were calculated by applying the 2^−ΔΔCt^ method [18].

### 2.3. Western Blot Analysis

For western blot analysis, cells were lysed in Laemmli sample buffer (500 mM Tris Cl pH 6.8, 30% Glycerol, 2% SDS, 0.01% Bromophenol Blue) and homogenized by sonication. Protein samples were boiled for 5 min at 100 °C, reduced with β-mercaptoetanol and separated by SDS-PAGE. The antibodies used in this study are the following: anti-β catenin, anti phospho β-catenin (S33/37/T41), anti Gsk3, anti phospho GSK3 (S9), anti actin, anti osterix, anti-osteocalcin, anti-Runx2, anti-Mepe (all from Santa Cruz, CA, USA). Mouse and rabbit HRP-conjugated secondary antibodies for western blot were purchased from GE Healthcare Europe GmbH.

### 2.4. Alizarin Red Assay

Matrix mineralization was evaluated by Alizarin Red-S (AR-S) staining. The cells were fixed in 4% Paraformaldehyde for 10 min at room temperature. After this time, fixative solution was removed, cultures were washed in PBS and plates were stored in PBS at 4 °C until sample processing. PBS was removed from the stored plates and the cell layer was stained with 2% Alizarin-S (Sigma-Aldrich, St Louis, MO, USA) at ~pH 4.2 for 5′. Cell preparations were washed with PBS to eliminate non-specific staining. To quantify calcium deposition, the dye was removed from the monolayer by the addition of 10% cetylpyridinium chloride solution until all the dye had been drawn from the monolayer. A volume of 200 μL of the solution (in duplicate) was transferred to a clean 96-well plate. Optical density was measured by spectrophotometry at 570 nm, using 10% cetylpyridinium chloride as a blank reference.

### 2.5. Immunofluorescence

MLO-Y4 cells were fixed on glass slides in 4% paraformaldehyde in PBS and permeabilized with 1% Triton X-100 in PBS for 10 min.

After a treatment with 3% BSA in PBS for 30 min at room temperature, samples were incubated with the primary antibodies diluted in PBS containing 3% BSA (rabbit anti-β-catenin diluted 1:20, overnight at 4 °C). After washing in PBS containing 3% BSA, the samples were incubated for 1 h at room temperature with the secondary Abs diluted 1:200 in PBS containing 3% BSA (goat anti-rabbit 488). After washing in PBS, the samples were mounted with anti-fading medium (0.21 M DABCO and 90% glycerol in 0.02 M Tris, pH 8.0). Negative controls consisted of samples not incubated with the primary antibody.

## 3. Results and Discussion

### 3.1. GSK3 Inhibitor CHIR99021 Activates Wnt/β-Catenin Signalling Axis in Osteocytes MLO-Y4

**Figure 1:**
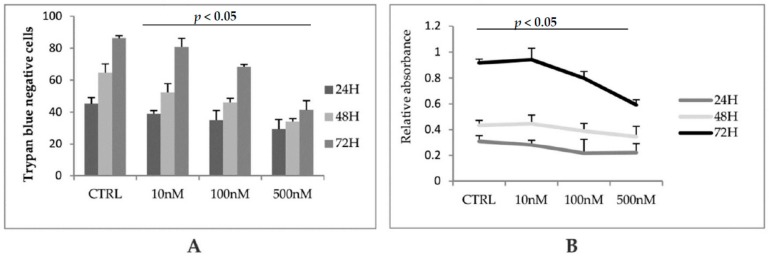
(**A**) Cells viability was measured by counting the unstained cells with Trypan blue. Where indicated cells were treated with 10 nM, 100 nM, and 500 nM of CHIR99021 for 24, 48, and 72 h. (**B**) The same samples were analyzed by 3-(4,5-dimethylthiazol-2-yl)-2,5-diphenyltetrazolium bromide (MTT) assay. The graph shows the relative absorbance read at 570 nM.

Glycogen synthase kinase 3 (GSK3), is a serine/threonine kinase with a pivotal role as key regulator of numerous signalling pathways. In particular, GSK3 has been found to be involved in multiple cellular processes including the Wnt pathway. In the activation of the canonical Wnt pathway, inhibition of GSK3 results in dephosphorylation of β-catenin leading to its nuclear accumulation. The role of GSK3 and Wnt in the differentiation of osteoblast cells has been previously described but the results are often in contrast each other and the molecular network has not been clarified sufficiently. It has been shown that activation of the Wnt pathway, via inhibition of GSK3, stimulates osteoblast differentiation [19], and that sclerostin itself could activate PDGFR inhibiting osteoblast differentiation. [20] On the other hand, it was found that GSK3 gain-of-function promotes in vitro osteogenesis of adipose-derived stromal cells [21], and other researchers established that GSK3β-deficient mice displayed an increment in bone formation due to enhanced expression and activity of the transcription factor RUNX2 [22]. For the first time, we analyzed the effects of GSK3 inhibitor CHIR99021 in terms of viability, cell cycle and downstream signaling. Firstly, we investigated whether GSK3 inhibition, obtained with CHIR99021 treatment, could cause effects in terms of reduced cell viability in osteocytes cell line MLO-Y4. By means of MTT metabolic assay and Trypan blue staining, we evaluated the viability of cells treated with different concentrations of the GSK3 inhibitor. The graphs shows that the treatment does not affect in statistical significance manner cells viability upon treatment for 24, 48 and 72 h in Figure 1A,B.

**Figure 2:**
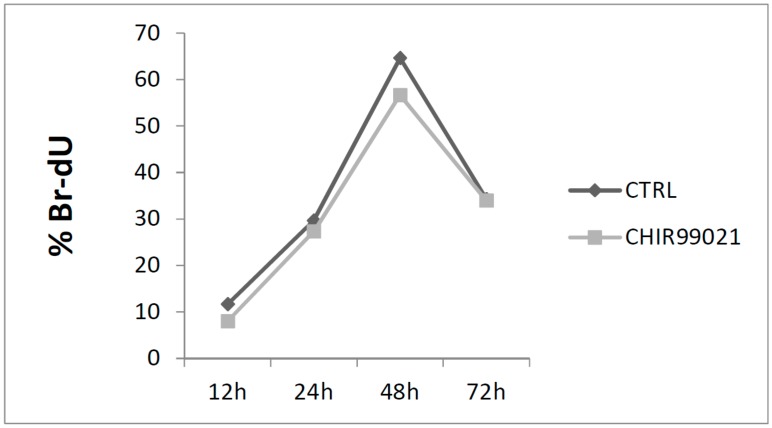
MLO-Y4 cells were treated with CHIR99021 at 100 nM for 12, 24, 48, and 72 h. BrdU staining was evaluated by Fluorescence-Activated Cell Sorter (FACS). Error bars represent ± ST.DEV (Standard Deviation).

To understand the effects mediated by CHIR99021 on cell cycle, BrdU incorporation was used to examine the rate of cell proliferation. The incorporation of BrdU during DNA synthesis was detected with GFP conjugated anti-BrdU antibody, and the percentage of cells in S phase was analyzed by flow cytometry. In both CHIR99021-treated and control cultures, the rate of cell proliferation decreased as the cultures moved from the pre-confluent to the post-confluent stage. Once the cultures became confluent, the rate of proliferation stabilized and effects mediated by treatment are not visible in Figure 2.

**Figure 3:**
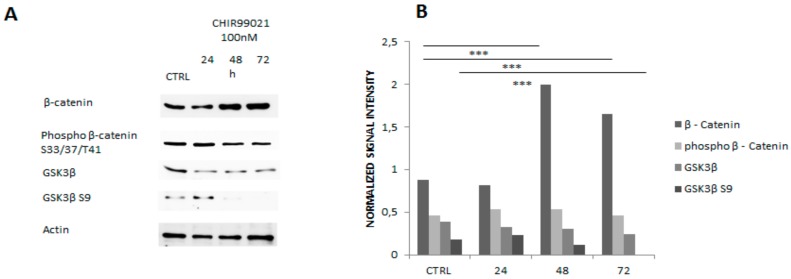
(**A**) 40 ug of MLO-Y4 protein extracts were separated in SDS-PAGE and evaluated by western blot for the indicated antibodies. (**B**) Optical density relative to three different experiments was measured and represented in the bars chart. *** *p* < 0.05.

To further assess the effect of CHIR99021 on its direct targets, we monitored the expression of β catenin and Gsk3 and their phosphorylation state, indicating their activation or inhibition, respectively. Immunoblotting analysis confirmed that endogenous Gsk3 was robustly dephosphorylated upon 48 and 72 h of CHIR99021 treatment in our model as also demonstrated by several studies [23,24,25] in Figure 3A,B.

**Figure 4:**
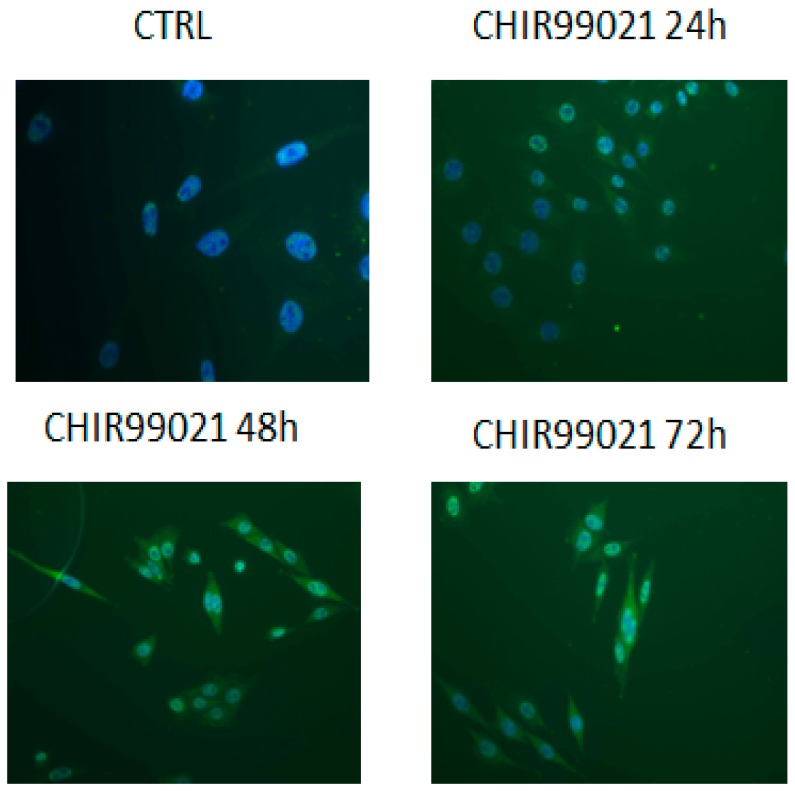
Cells treated with CHIR99021 at 100 nM for 24, 48, and 72 h were stained with β-catenin antibody and revealed by FITC(Fluorescein-5-isothiocyanate)conjugated secondary antibody. Magnification is 40×.

**Figure 5:**
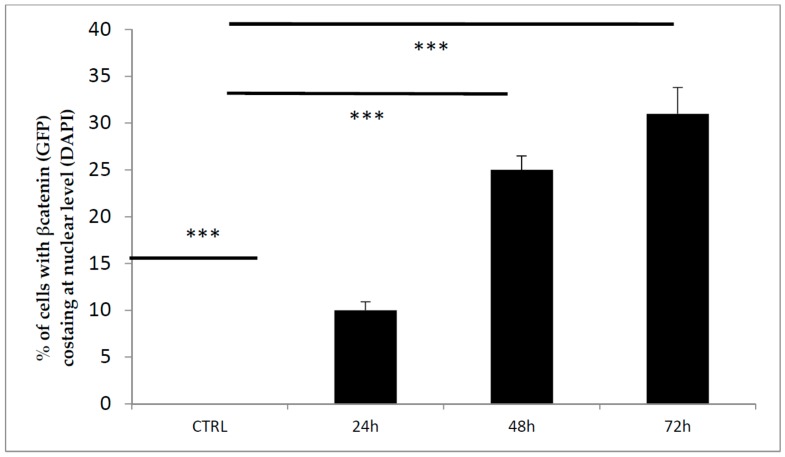
The bars chart shows the percentage of cells expressing Green fluorescent protein (GFP) at nuclear level normalized on total cell number revealed by DAPI staining. *** *p* < 0.05.

**Figure 6:**
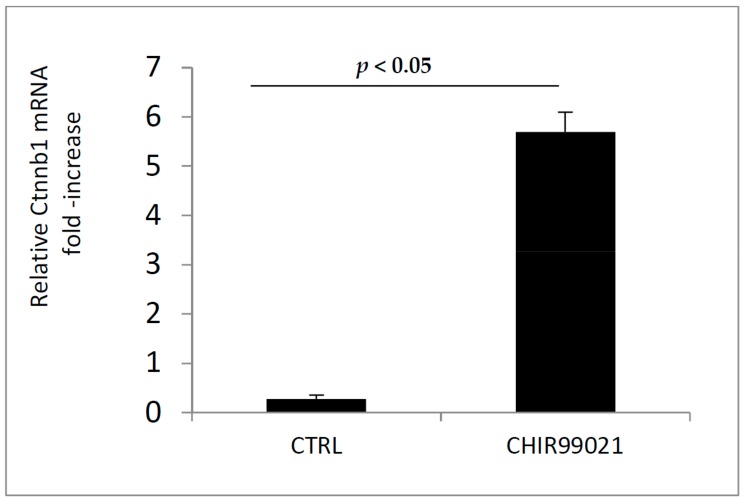
mRNA was extracted from MLO-Y4 cells treated or not with CHIR99021 at 100 nM for 48 h. The expression of β catenin gene (Ctnnb1) was evaluated by RT-PCR.

In parallel, pharmacologic inhibition of GSK3 activity can lead to stabilization of β-catenin. Indeed, we found that the protein levels of β-catenin in the nucleus were significantly higher in CHIR99021-treated cells than in vehicle control cells (Figure 4 and Figure 5). The same results were also confirmed by quantitative PCR (Figure 6).

**Figure 7:**
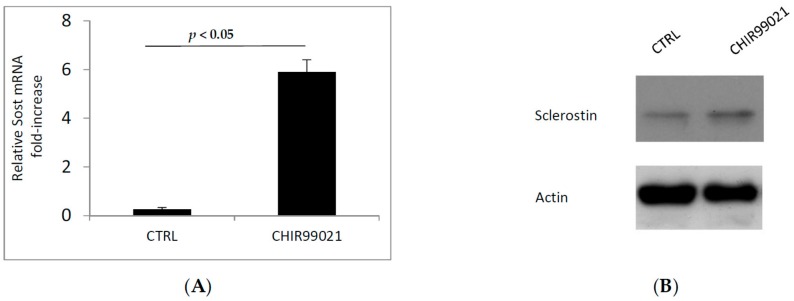
(**A**) mRNA was extracted from MLO-Y4 cells treated with CHIR99021 at 100 nM for 48 H. The expression of Sclerostin gene (Sost) was evaluated by RT-PCR. (**B**) A replicate of same samples were extracted for immunoblotting procedure. 40 μg of protein extracts were separated in SDS-PAGE and Sclerostin protein was revealed.

Then, we examined whether the GSK3 inhibitor could modulate the expression of Sclerostin, one of the most important inhibiting factor of bone formation. By the use of RT-PCR and immunoblotting, we analyzed first the level of Sost mRNA and second the expression of Sclerostin protein in MLO-Y4 cells treated or not with CHIR99021 (Figure 7A,B). Consistent with low level of Sclerostin in MLO-Y4 cells [26,27] the exposure of CHIR99021 can significantly increase the expression levels of Sclerostin compared to the vehicle control.

### 3.2. GSK3 Inhibitor Up-Regulate Osteogenesis Specific Genes Dependent on Wnt/β-Catenin Signaling

**Figure 8:**
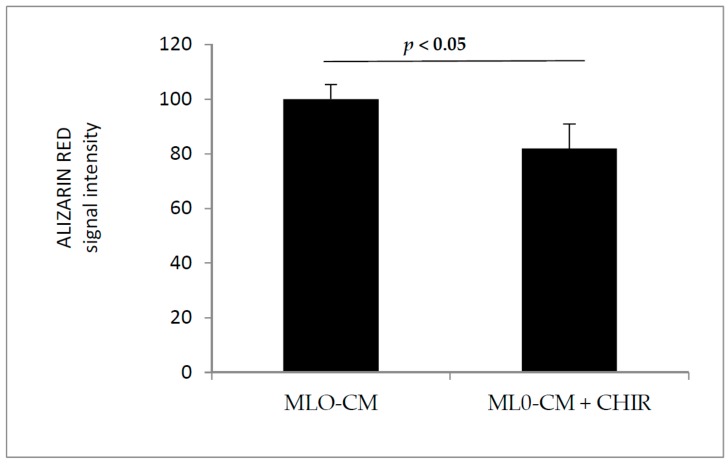
2T3 osteoblast cells were maintained in differentiation medium for 5 days. The differentiating medium was added in a ratio of 1:4 with medium from MLO-Y4 that were previously treated with CHIR99021 at 100 nM for 48 h. At the end of the differentiation procedure, 2T3 were stained with Alizarin Red and the absorbance was read at 405 nM. The bars chart represents the normalized value of Alizarin Red signal intensity calculated from three independent experiments.

**Figure 9:**
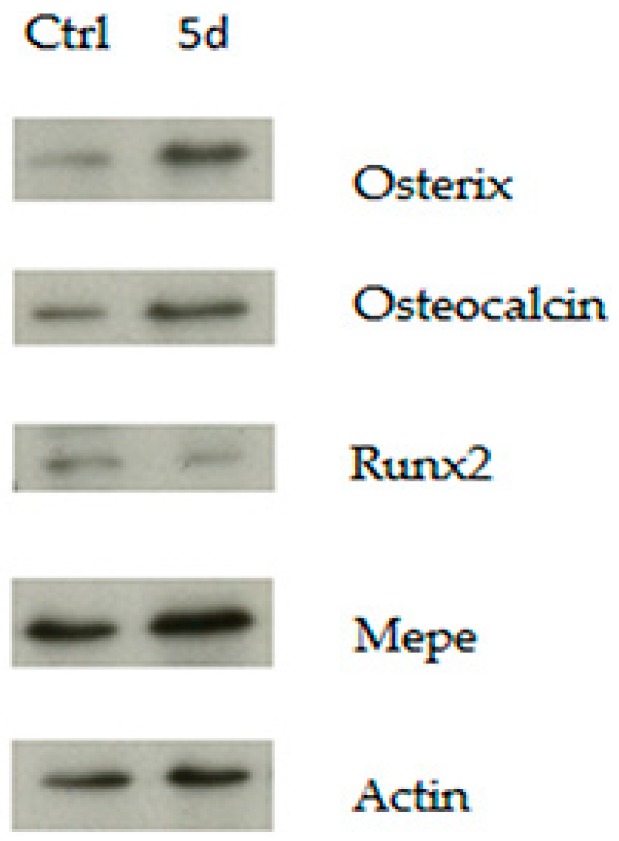
30 μg of protein extracts derived from 2T3 osteoblasts maintained in cycling (Ctrl) and differentiating condition (5d) for 5 days were separated in SDS-PAGE and the indicated proteins were revealed by immunoblotting.

In addition to regulating the local bone remodeling, the osteocytes are considered as endocrine cells, controlling phosphate homeostasis, bone matrix mineralization, and muscle-bone cross talk [28]. As such, osteocytes are emerging targets for drugs companies aimed at controlling the release of proteins that regulate bone metabolism [29]. To investigate whether the factors produced by osteocytes were able to modulate the physiological differentiation process of osteoblasts, we conditioned 2T3 osteoblast cells during differentiation with MLO-Y4 medium. We compared by functional assay, such as Alizarin Red, the effect of the activation of Wnt signaling by GSK3 inhibition. The results showed that conditioned medium obtained by MLO-Y4 cells treated with CHIR99021 was able to decrease the amount of mineralization produced by 2T3 upon 5 days of differentiation (Figure 8). Western blot assay with the indicated antibodies demonstrated the success of differentiation procedure (Figure 9).

**Figure 10:**
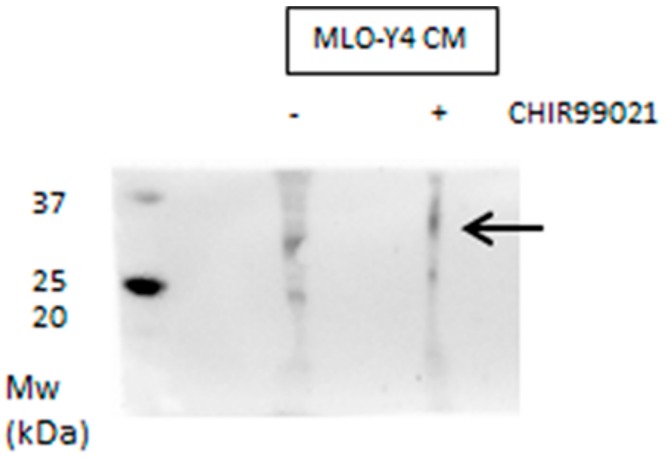
conditioned media from MLO-Y4 treated or not with CHIR99021100 nM for 48 h were concentrated and analyzed in SDS-PAGE. Sclerostin antibody revealed a band relative to 27–30 kDa.

Based on these findings we analyzed the conditioned medium of MLO-Y4 treated or not with CHIR99021 for the expression of Sclerostin. Media obtained by osteocytes were concentrated and analyzed in monodimensional electrophoresis. The expression of Sclerostin increased with exposure to GSK3 inhibitors and was secreted consistently in the growing medium (Figure 9 and Figure 10), suggesting that GSK3 inhibitor induces definitive modulation of osteoblast differentiation depending on the activity of canonical Wnt/β-catenin signaling in osteocytes.

**Figure 11:**
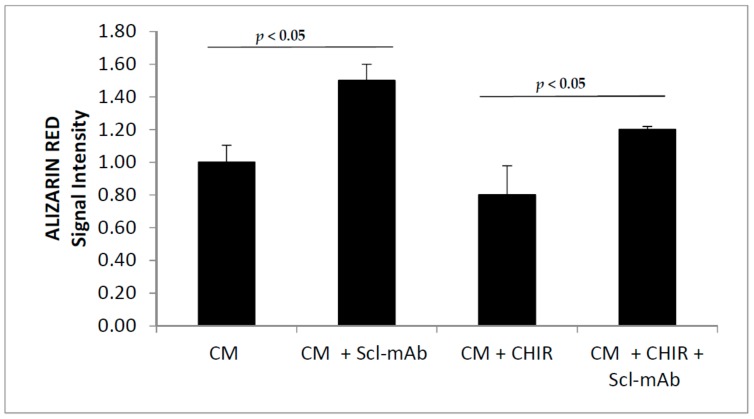
2T3 osteoblast cells were maintained in differentiation medium for five days. The differentiating medium was added in a ratio of 1:4 with medium from MLO-Y4 (CM) that were previously treated with CHIR99021 (CM + CHIR) at 100 nM for 48 h. Where indicated, MLO-Y4 media were pre-treated with Sclerostin antibody for 3 h at 37 °C (Scl-mAb).

In order to associate the effect of diminished mineralization ability of 2T3 conditioned with MLO-Y4 medium upon treatment with CHIR99021, we inactivated secreted Sclerostin with a specific antibody, and we conditioned 2T3 differentiation. Figure 11 shows the alizarin red amount of 2T3 osteoblasts differentiated for five days and conditioned with MLO-Y4 medium. Sclerostin inactivation obtained through neutralization with specific antibody, is able to revert the 2T3 capability to differentiate.

At the end of the differentiation procedure, 2T3 were stained with Alizarin Red, and the absorbance was read at 405 nM. The bar chart represents the normalized value of Alizarin Red signal intensity calculated from three independent experiments.

In conclusion, using a model of osteocyte and osteoblast cell lines, our paper demonstrated that GSK3 inhibition in osteocytes, resulting in a hyper activation of Wnt signaling, induces an increase in the production of Sclerostin, responsible as a soluble factor, of the decreased capability of osteoblast to produce mineralized matrix in differentiating conditions.
